# Recent Advances in Graphene Adaptive Thermal Camouflage Devices

**DOI:** 10.3390/nano14171394

**Published:** 2024-08-26

**Authors:** Lucia Sansone, Fausta Loffredo, Fabrizia Cilento, Riccardo Miscioscia, Alfonso Martone, Nicola Barrella, Bruno Paulillo, Alessio Bassano, Fulvia Villani, Michele Giordano

**Affiliations:** 1 Institute for Polymers, Composites and Biomaterials, National Research Council of Italy (CNR), 80055 Portici, Italy; lucia.sansone@cnr.it (L.S.); alfonso.martone@cnr.it (A.M.); michele.giordano@cnr.it (M.G.); 2 Nanomaterials and Devices Laboratory, Italian National Agency for New Technologies, Energy and Sustainable Economic Development (ENEA), 80055 Portici, Italy; fausta.loffredo@enea.it (F.L.); nicola.barrella@enea.it (N.B.); fulvia.villani@enea.it (F.V.); 3 Leonardo Innovation Labs, Quantum Technologies, Optronics and Materials Lab, Via Albert Einstein 35, 50013 Campi Bisenzio, Italy; bruno.paulillo@leonardo.com; 4 Leonardo Electronics, Defence Business Area, Via Valdilocchi 15, 19136 La Spezia, Italy; alessio.bassano@leonardo.com; 5 CRdC Tecnologie Scarl, Via Nuova Agnano 11, 80125 Napoli, Italy

**Keywords:** camouflage, nanomaterials, emissivity, infrared

## Abstract

Thermal camouflage is a highly coveted technology aimed at enhancing the survivability of military equipment against infrared (IR) detectors. Recently, two-dimensional (2D) nanomaterials have shown low IR emissivity, widely tunable opto-electronic properties, and compatibility with stealth applications. Among these, graphene and graphene-like materials are the most appealing 2D materials for thermal camouflage applications. In multilayer graphene (MLG), charge density can be effectively tuned through sufficiently intense electric fields or through electrolytic gating. Therefore, MLG’s optical properties, like infrared emissivity and absorbance, can be controlled in a wide range by voltage bias. The large emissivity modulation achievable with this material makes it suitable in the design of thermal dynamic camouflage devices. Generally, the emissivity modulation in the multilayered graphene medium is governed by an intercalation process of non-volatile ionic liquids under a voltage bias. The electrically driven reduction of emissivity lowers the apparent temperature of a surface, aligning it with the background temperature to achieve thermal camouflage. This characteristic is shared by other graphene-based materials. In this review, we focus on recent advancements in the thermal camouflage properties of graphene in composite films and aerogel structures. We provide a summary of the current understanding of how thermal camouflage materials work, their present limitations, and future opportunities for development.

## 1. Introduction

Thermal camouflage technology seeks to lower an object’s detectability and identification by reducing its emitted infrared (IR) radiation (i.e., IR signature), thereby achieving IR stealth [1,2,3,4,5]. The goal is to match the apparent temperature of an object, as detected by an IR camera, with that of the background to reduce its observability by an IR imaging system. This is achieved by lowering the thermal contrast by minimizing the difference between the apparent temperature of the object’s surface and the background as much as possible by modulating its thermal emissivity and/or surface temperature [5,6,7,8,9,10]. In this way, the target object will appear blended into the background when observed by a thermal IR imager.

This review reports on the use of 2D graphene-like nanomaterials in thermal camouflage technology; in particular, it includes the recent literature based on multilayer graphene (MLG), graphite nanoplatelet (GNP) coatings, and graphene aerogel (GA) as dynamically adaptive materials for thermal emissivity modulation. It begins with an introduction to the principles of thermal camouflage, followed by a description of recent advancements in graphene and graphene-like materials for this application. Additionally, this review summarizes current limitations and future opportunities for the research and development of graphene-based thermal camouflage devices.

### 1.1. Thermal Camouflage Principle

**Figure 1:**
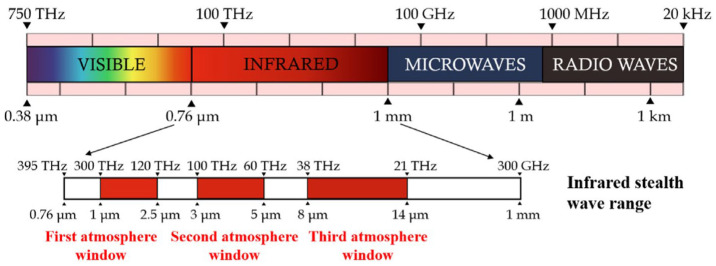
IR window and corresponding IR stealth wave range. Reprinted with permission from Ref. [13]. Copyright 2023 Elsevier.

Every object emits energy in the form of electromagnetic (EM) waves as a function of its temperature (i.e., thermal radiation): the higher the object’s temperature, the more energy it radiates overall. For objects having temperatures close to or above room temperature, thermally emitted radiation mostly falls in the infrared (IR) or visible spectra [10]. Depending on the wavelength, an object’s heat radiation is attenuated to varying degrees during its transmission in air. The term atmospheric window describes a spectral band where the atmospheric attenuation is low. In particular, three atmospheric windows are present in the IR range: the short-wave IR band (SWIR, 1–2.5 μm), the medium-wave IR band (MWIR, 3–5 μm), and the long-wave IR band (LWIR, 8–14 μm) (see Figure 1). Therefore, most IR detectors employed in military applications operate in these three bands. In particular, the LWIR band, where thermal infrared imagers and cameras are predominantly utilized [11,12], has been the focus of most studies on thermal cloaking techniques, specifically in the 8–14 μm range. According to the Stefan–Boltzmann law [10], the power radiated from an object’s surface across all wavelengths (*E*) is given by:

(1) $$E=\varepsilon \sigma T^{4}$$

where *ε* represents the object’s emissivity, *σ* represents the Stefan–Boltzmann constant, and *T* is the object’s thermodynamic temperature (measured in Kelvin, *K*). The emitted IR radiation power is directly related to the emissivity and the fourth power of the temperature of the target surface. Based on these principles, thermal camouflage strategies can be categorized into two types: low-emissivity material design and temperature-control material design.

According to the Stefan–Boltzmann law, to achieve thermal camouflaging of an object it is necessary to modify its IR emissivity and/or its surface temperature. The emissivity depends on the IR optical properties of the surface. Moreover, the emissivity equals absorbance according to Kirchhoff’s law and, owing to the energy conservation law, the relationship between the IR emissivity (*ε*), IR absorptivity (*α*), and IR reflectivity (*R*) for opaque materials (transmissivity *T* = 0) is [14]:

(2) ε=α=1−R

Therefore, by controlling the reflectivity of the material in the IR band, it is possible to control its emissivity. In 1903, Hagen and Rubens derived the relation between optical properties and the electrical resistivity of metals at sufficiently low frequencies (*ω* << 1/(*ρ*∙*ε*_0_)) [15]:

(3) $$\varepsilon =2\sqrt{2\omega \varepsilon_{0}\rho }$$

where *ε* is the normal spectral emissivity, *ω* is the angular frequency, *ε*_0_ is the dielectric constant of a vacuum, and *ρ* is the DC electrical resistivity of the reflective material (metal).

Following the formulation of the Hagens–Rubens relation, an extension of Equation (3) beyond the far-infrared region has been proposed by introducing a generalized formulation of Ohm’s law. Although this relationship does not accurately describe the behavior of all current-conducting materials, experimental works confirm a dependence between static electrical conductivity and their IR reflection coefficient. Based on such principle, a wide variety of conducting and resistive materials can potentially exhibit a dependence on the infrared emissivity from the conductivity. 

### 1.2. Thermal Camouflage Materials

In Table 1, the most important classes of materials used for thermal camouflage are reported and their advantages and disadvantages are summarized.

The simplest way to achieve thermal camouflage is through a passive system, typically using low-emissivity metallic layers made from powders of Al, Cu, Ag, and similar materials. Emissivity reduction can be controlled by adjusting the powder’s shape, roughness, thickness, and other factors [8,29]. These metallic-based coatings can minimize the difference between the thermal IR radiation of the target and the background, rendering them undetectable to monitoring systems [30,31]. However, these low-emissivity metallic powders have high reflectivity for electromagnetic fields, making them easily identifiable by radar detection [32,33]. Additionally, these metal powders can oxidize in the air, leading to higher IR emissivity and significantly reducing the coating’s thermal camouflage performance [16,34]. To avoid radar detection and oxidation of metal powders, the proportion of metal fillers should be decreased in organic polymer coatings [17,18,19]. However, this can also increase emissivity. 

While static camouflage is easy to apply, it has the drawback of fixed emissivity, meaning that targets can only be hidden at a constant background temperature. In practical situations, targets may move, or the background temperature may change; under such conditions, the static thermal camouflage performance will degrade, leading to detection due to inconsistent IR signatures. In contrast, dynamic thermal camouflage is more practical and promising for future military applications. This approach allows objects to quickly adapt to changing environments, increasing the likelihood of remaining undetected. Dynamic thermal camouflage requires adaptive materials that respond to external stimuli, such as electricity, light, temperature, moisture, mechanical forces, or vapor. Various strategies have been developed to engineer thermal emissivity, some of which are used to achieve dynamic thermal camouflage.

To enhance the performances of stealth materials, researchers introduced new thermal camouflage materials, including metal-oxide semiconductors, such as CeO_2_, ZnO, antimony oxides (ATO), indium tin oxide (ITO), etc. [20,21,27,28], metamaterials [35,36], and two-dimensional (2D) nanomaterials [37,38,39,40,41,42]. Metal-oxide semiconductors offer some IR stealth capabilities [37,38], but their use is limited by high IR emissivity, toxicity, and production cost [39,40,43]. Metamaterials perform well in camouflage applications, but their complex designs make manufacturing difficult. New nanofabrication techniques have enabled the deposition of large-area noble metal films (e.g., Ag, Cu, and Au) only a few nanometers thick, allowing for an optimal balance between electrical conductivity and optical transparency [24,25,26]. Recently, novel low-emissivity 2D materials have been employed as passive systems to achieve thermal camouflage, such as MXenes (Ti_3_C_2_T_x_). Similar to metal particles, these materials are employed as micrometer-thick films, guaranteeing low IR emissivity of 0.17–0.2, but they are lightweight and have microwave absorption capacity. In addition, they are slightly sensitive to high temperatures, maintaining an effective cooling effect. However, the surface morphology, structure, and functional groups of these materials influence the thermal camouflage ability and stability. Appropriate synthesis methods are needed to adjust the surface defect, the size, and the type of functional groups of MXenes [13]. On the contrary, graphene-based materials have a high emissivity of about 0.6. Compared with the previously mentioned materials, graphene and graphene-like materials are the most appealing for thermal camouflage due to their ability to dynamically adjust emissivity [38,39]. 

In active or adaptive thermal camouflage systems, the infrared signature of an object is modified to match the background dynamically. According to Equation (1), this can be obtained by modulating dynamically either the temperature or the emissivity [44]. The latter strategy allows the modification of the apparent temperature of the target object by altering IR optical properties and has been demonstrated to be highly promising [45,46,47,48,49].

For this purpose, so-called chromogenic materials with dynamically tunable IR optical properties upon external stimulation (e.g., voltage, temperature, illumination, etc.) have been employed in the literature for fabricating thermal camouflage devices. Electrochromic (EC) materials offer a practical way to reconfigure optical properties (e.g., IR absorbance/emissivity in the case of thermal camouflage) by using an inexpensive electrical control, such as a low bias voltage. Conventional EC materials include inorganic and organic layers, such as metal oxides (e.g., WO_3_) or conductive polymers (e.g., PANI) developed over decades and today embedded in commercial products, such as smart windows [22]. More recently, innovation in nanotechnology pushed frontiers in EC materials owing to the rise of ultrathin and two-dimensional (2D) materials, such as ultrathin metals, graphene, and related materials. For example, quantum wells (QWs) based on heterostructured semiconductors (e.g., GaAs/AlGaAs) provide 2D confinement and can be engineered to show tunable IR absorption at desired IR wavelengths upon biasing [23]. However, optical transitions in QWs yield a narrow band response, which is undesirable for managing thermal radiation in the MWIR and LWIR bands.

By exploiting the nanoscale manipulation of metals, Liu et al. proposed a highly performing IR adaptive EC surface based on reversible metal electrodeposition (RMED), where an ultrathin Ag film can be reversibly deposited/dissolved over a (semi)transparent electrode upon biasing. The IR surfaces are devised to switch from a high-reflectance to a high-absorbance/emissivity state, yielding a very large emissivity modulation [25]. The same technology based on Cu deposition has recently shown the highest emissivity modulation reported so far, close to 0.85 [24]. Another freshly proposed approach consists of the direct modulation of the IR properties of ultrathin metals by promoting a reversible nanoscale reduction/oxidation process via electrolyte gating [26]. However, such metal-based active surfaces suffer from several drawbacks to be fixed, such as the slow response time (several seconds) associated with RMED or redox processes, their limited lifetime, and cyclability. 

Remarkably, graphene and related 2D materials have emerged as the most promising material platforms for IR adaptive surfaces due to their highly customizable electro-optical properties that can be adjusted upon electrostatic or electrochemical gating. Graphene is a single layer of sp^2^-bonded carbon atoms arranged hexagonally [50,51]. The sp^2^ hybridization and the very thin atomic thickness [52] confer graphene with high strength [53,54], high electricity [55], and heat conduction [56,57,58]. It is a zero-band-gap semiconductor featuring broadband optical transparency, mechanical flexibility, tunable ambipolar electrical conduction, and high carrier mobility, which made it a popular material for applications in transparent electronics, optical detectors, modulators, and wearable sensors, to name a few. In particular, the band structure of graphene allows for the easy control of the Fermi energy/carrier density through electrical gating using thin-film dielectrics or electrolyte media and/or chemical doping, which results in widely tunable electrical conductivity and optical properties at mid-IR wavelengths [59,60]. All these properties refer to an ideal material, since producing monolayer graphene sheets without defects, such as with chemical vapor deposition (CVD), is challenging and highly expensive [61]. 

However, the IR absorption of single-layer graphene is relatively small, which is insufficient to achieve large emittance modulation. Multilayer graphene and graphene nanoplatelets, comprising tens or hundreds of layers, related nanocomposites, and/or aerogels were shown instead to have larger absorption/emissivity that can be significantly tuned upon electrolyte gating, as we will discuss below, also affording a good compromise between low cost and performance [62].

## 2. Adaptive Thermal Camouflage Devices Based on Graphene Materials

As mentioned before, one promising approach for adaptive thermal camouflage devices is based on reducing the thermal radiation emitted by an object via the modulation of surface emissivity. Graphene-based materials have been demonstrated to be suitable for this application owing to the possibility of modulating their emissivity by electrical gating. Specifically, recent literature reports the use of multilayer graphene produced by the chemical vapor deposition method, graphite nanoplatelet coatings, and graphene aerogels as effective materials for adaptive thermal camouflage applications.

For the first time, Salihoglu et al., 2018 [41] demonstrated the capability for tuning the thermal radiation of multilayer graphene (MLG) grown by chemical vapor deposition (CVD) by controlling its emissivity through electrolyte gating.

### 2.1. General Architecture and Principal Elements

**Figure 2:**
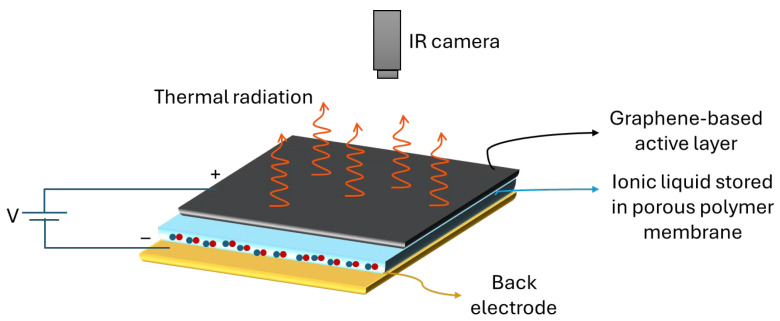
Adaptive thermal camouflage scheme and device functioning.

The architecture of a typical adaptive thermal camouflage device, similar to the one first proposed by Salihoglu et al., 2018 [41], is schematized in Figure 2. As thermal radiation originates from the very top surface, the dynamic control of the device emissivity is obtained by electrically gating the active layer using a non-volatile room-temperature ionic liquid (RTIL) that intercalates into the graphene layers when a bias voltage is applied. In this way, the electrical conductivity of the active layer is modulated and the energy levels change, inducing a tunable IR optical absorption. 

In detail, the device consists of a graphene-based electrode stacked on a porous membrane soaked in an RTIL and a back electrode. By applying a voltage bias to the device, the ions contained in the porous membrane dope the active graphene layers, resulting in an intercalation phenomenon. The charge density and Fermi energy of the graphene layer are modified and, as a result, the emissivity of the device can be dramatically decreased, eventually yielding thermal camouflage functionality. Below, we illustrate the function of each layer contained in the device. 

#### 2.1.1. Back Electrode

The function of the back electrode is to prevent the transmission of the background thermal radiation and to serve as the gate electrode. 

#### 2.1.2. Porous Membrane and Ionic Liquid

The purpose of the membrane is to physically separate the positive and negative electrodes in order to avoid shorts and to support the ionic liquid while, at the same time, permitting an almost free ionic movement.

The most used membranes in the literature are those used as separators in liquid electrolyte Li-ion batteries (Table 2). Specifically, thin polymeric membranes are widely used as separators, thanks to their low thickness that guarantees the high volumetric energy density of Li-ion batteries. The separator function is critical in liquid electrolyte batteries since it should prevent physical contact between the positive and negative electrodes while allowing free ionic transport and isolating electronic flow [63].

Such microporous layers must be chemically and electrochemically stable and mechanically strong, and must have the right level of porosity to absorb liquid electrolytes to yield high ionic conductivity. The pore size should be at least of the same order as the ion size to allow the desired ionic conductivity between the electrodes, since smaller pore sizes could block the migration of particles from one electrode to the other [64].

Common separators used in batteries and employed for the fabrication of thermal adaptive camouflage are Celgard membranes. These are polypropylene (PP)/polyethylene (PE) membranes with 38 µm thickness and 45% porosity that have demonstrated good electrolyte wettability [65]. 

Room-temperature ionic liquids (RTILs) employed as intercalants to tune the physical properties of graphene active layers are solvents with unique characteristics: they are liquid salts at room temperature (melting points < 100 °C) and, unlike traditional organic solvents, are characterized by low volatility, high ionic conductivity, high charge density and high stability over a large electrochemical window, being thermally and chemically stable, even under extreme conditions. These properties make them suitable for use as electrolytes in applications that require high voltages, i.e., energy-storage devices, batteries, and capacitors [66]. Moreover, in the form of ionic gels, they have been employed to modulate the electro-optical properties of several emerging ultrathin materials (single-layer graphene, ultrathin metals, etc.) owing to their very high specific capacitance. 

RTILs often consist of an organic cation interacting with an inorganic anion. The most common cations found in ionic liquids are quaternary ammonium and imidazolium salts, while the anions can be of an inorganic or organic nature. Yu et al. [67] investigated the effect of the intercalation of 44 different ionic liquids, demonstrating that the intercalation of [TFSI]^−^ anions provides the best performance in terms of emissivity modulation and long-term stability for adaptive IR camouflage surfaces. The threshold voltage for intercalation in an ionic liquid is influenced by the size difference between anions and cations. Interestingly, when a large ion is paired with a smaller counterion, the required threshold voltage is relatively low. However, when the ion sizes are comparable, the threshold voltage is likely determined by the interaction of the ions with the surface, (i.e., interface capacitance).

#### 2.1.3. Graphene-Based Active Layers

Several active layers based on graphene and related nanostructures, such as multiplayer graphene (MLG), aerogels (AGs), and Van der Waals (VdW) nanolayers, have been used in the literature for the dynamic control of thermal radiation. 

**Figure 3:**
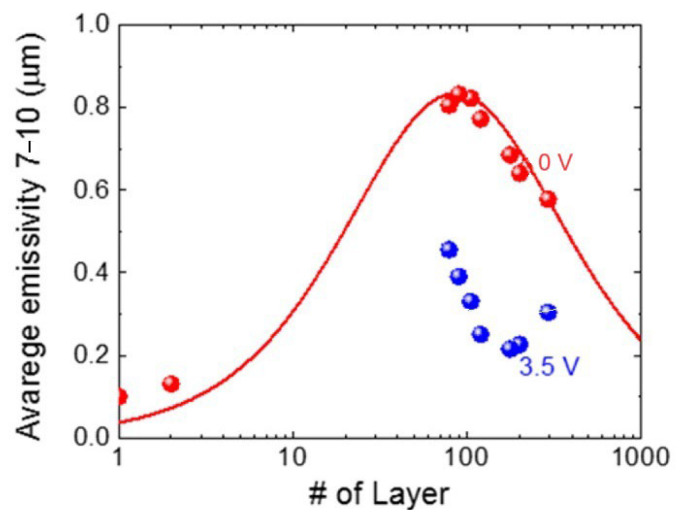
Emissivity’s dependence on the number of graphene layers. Reprinted with permission from Ref. [41]. Copyright 2018 American Chemical Society.

Graphene shows broadband optical absorption due to its linear band dispersion. For monolayer graphene, the optical absorption is limited to 2.3% in the visible and near-IR regions, where interband transitions dominate, whereas intraband and disorder-mediated processes set the optical response in the far and mid-IR bands. Specifically, the optical conductivity of graphene in the thermal IR region depends on the Fermi energy, which can be tuned by doping. To modulate the graphene emissivity/optical absorbance, it is necessary to shift the Fermi level to a high energy level. For the MLG used as a prototype material to build adaptive IR surfaces, it was found that a stack with several layers of graphene can be considered optically equivalent to a graphene layer with carrier density given by the sum of those of the individual layers [68]. Experiments on MLG stacks for adaptive IR surfaces have shown a maximum emissivity/absorptivity of ≈0.8 for a stack of ≈100 layers (Figure 3). Thicker or thinner films yield less emissivity due to higher reflectivity or smaller absorption, respectively [41]. Thus, the thickness of the graphene-based active layer (MLG or other) and, overall, the as-fabricated conductivity/doping level are important parameters that define the achievable modulation range of the emissivity. Furthermore, the emissivity of the pristine material should be neither too high nor too low. An initial high emissivity implies a low electrical conductivity, which would not allow the movement of charges. On the contrary, an initial low emissivity would not allow sufficient dynamics for the modulation of the final emissivity.

In the following paragraphs, we will present the main architectures and results on graphene-based adaptive surfaces for thermal camouflage.

### 2.2. Thermal Camouflage Devices Based on MLG Active Layer

**Figure 4:**
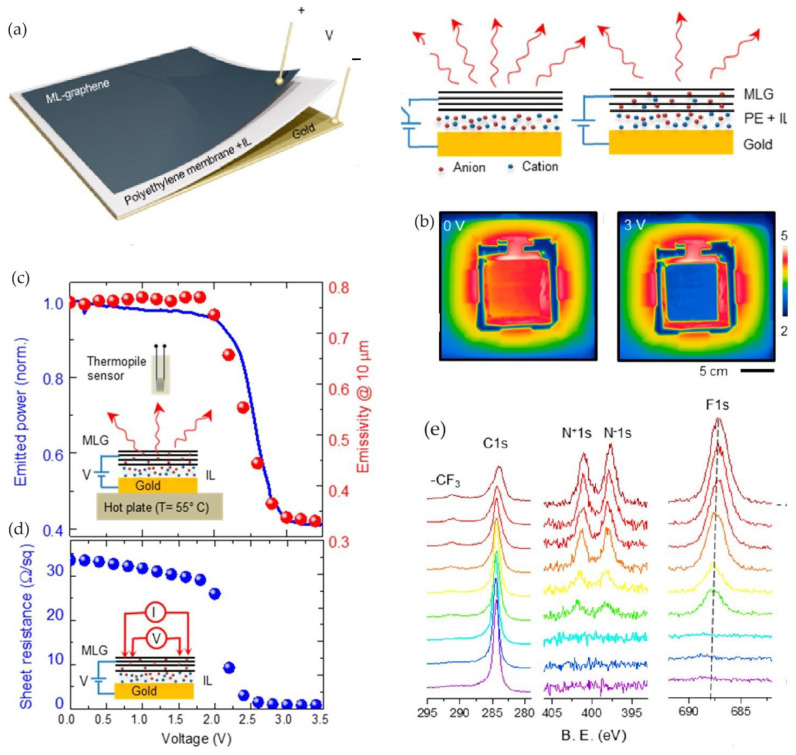
MLG-based device by Salihoglu et al. [41]. (**a**) Schematic of the active thermal surface and working principle (the red arrows refers to the emitted radiation); (**b**) thermal images of the device at bias voltages of 0 and 3 V; (**c**) emitted thermal power and extracted emissivity versus voltage at 10 μm; (**d**) sheet resistance of the ML graphene electrode versus bias voltage; (**e**) XPS spectra recorded from the surface of the device under bias voltage between 0 and 4 V. Adapted with permission from Ref. [41]. Copyright 2018 American Chemical Society.

Salihoglu et al. studied the first example of a thermal camouflage device based on a multi-layer graphene (MLG) layer [41]. They fabricated an innovative device featuring a gating scheme that intercalates an ionic liquid into graphene layers, resulting in significant tunable emissivity in the IR region. The device consisted of an MLG electrode stacked on a porous polyethylene (PE) membrane infused with the ionic liquid N,N-diethyl-N-(2-methoxyethyl)-N-methylammonium bis-(trifluoromethylsulfonyl)imide (DEME-TFSI), and a back gold electrode (Figure 4). The PE membrane was IR transparent and held the electrolyte.

The MLG active thermal surface demonstrated efficient real-time electrical control of thermal emission across the IR spectrum without changing the surface temperature. The device operated in the bias range of 0–3.5 V, showing excellent modulation of emissivity between 0.76 and 0.33 at 10 μm wavelengths. This effect was detected by an IR camera as a reduction in the apparent temperature of the object (Figure 4). The thermal radiation emitted by the device mainly originated from the top graphene electrode, given the very low emissivity of the gold substrate (<0.01) and the IR transparency of the PE membrane.

To quantify the performance of the active thermal surface, the device was heated at 55 °C, and a different bias voltage between 0 and 3.5 V was applied. Thermal images showed significant variation in thermal appearance across the entire device area (Figure 4). At high voltages, the apparent temperature of the MLG surface significantly reduced (by about 15 °C, Figure 4) due to reduced emissivity, making it appear as cold as the gold back electrode. The variations in total radiated power and extracted emissivity values showed similar voltage dependence, indicating a nearly constant emissivity variation over the mid-IR range with bias voltage changes. 

The change in emissivity was accompanied by a change in electrical resistance. The sheet resistance of MLG, measured by the four-point resistivity method, showed the same behavior as emissivity, with a switch at a voltage bias of 1.5 V. The sheet resistance dropped from 33 Ω/sq to 0.6 Ω/sq. The authors attributed the reduction in emissivity (and sheet resistance) to graphene doping caused by ionic liquid intercalation. XPS characterization of the active thermal surfaces quantified the intercalation process. XPS spectra recorded from the device surface at bias voltages between 0 and 4 V (Figure 4d) revealed the appearance of N 1s and F 1s peaks, typical of the ionic liquid, for gatings above 1.5 V, indicating the onset of intercalation and the threshold voltage.

The intercalation process is reversible, allowing the device to switch between high and low emissivity values with a time constant of 0.5 s and a response time of less than 1 s for multiple cycles. The authors noted a slight shift in the threshold voltage due to hysteresis in the intercalation process.

The MLG active thermal surface demonstrated an efficient real-time electrical control of thermal emissions over the full infrared (IR) spectrum without changing the temperature of the surface. 

**Figure 5:**
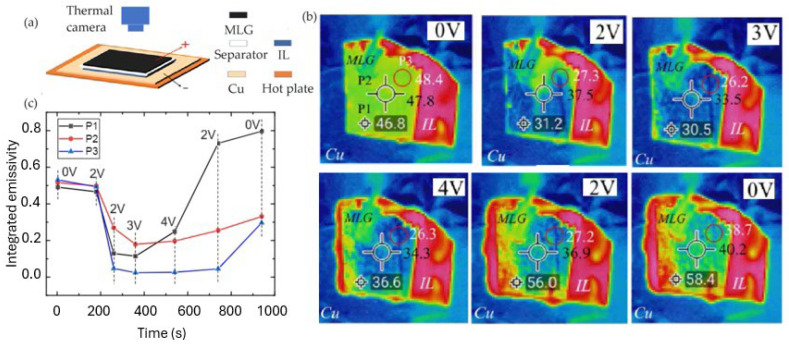
Huang et al. [69]. Schematic structure of device (**a**). Apparent temperature variation by IR camera for [EMIm][NTf_2_] (**b**) and emissivity modulation in different control points (**c**). Adapted with permission from Ref. [69]. Copyright 2021 American Chemical Society.

Huang et al. [69] investigated a variety of ILs characterized by different anions and cations for modulating the emissivity of MLG. They fabricated an infrared tunable device using MLG as the active layer, a Celgard 2325 separator (25 μm thick, polypropylene–polyethylene–polypropylene copolymer), and a Cu back electrode. They used 20 μL of IL by injecting it between the separator and back electrode (Figure 5). Six different ILs have been tested; specifically, two sets of ILs with anions of different sizes have been employed: [EMIm][NTf_2_], [HMIm][NTf_2_], [PhCH_2_MIm][NTf_2_], [BMIm][BF_4_], [HMIM][BF_4_], and [EMIm][BF_4_]. 

The modulation depth of emissivity, which is defined as the difference between the initial highest and final lowest emissivities, is modified according to the ILs used. The best modulation depth was found for [EMIm][NTf_2_], with a reduction in the emissivity from 0.54 to 0.02. The apparent temperature of the MLG surfaces reduces with the applied voltage by about 10 °C. However, the highest lifetime was found for [EMIm][BF_4_]. The authors demonstrated that the intercalation of a large-size ion ([NTf_2_]^−^) results in the severe and irreversible degradation of MLG, while small size-ions ([BF_4_]^−^) maintain the structural integrity of MLG, also increasing the device lifetime.

Cui et al., 2022 [70] realized an etchable cloth substrate that could produce a large-area free-standing graphene cloth film made of papyrus (FS-GFF) with high conductivity and favorable maneuverability to be employed as the active layer for fabricating an adjustable infrared camouflage textile device. The electrical conductivity of FS-GFF varies according to the thickness of the graphene layer in the range of 50 Ω sq^−1^ (at 90 nm) to 2800 Ω sq^−1^ (at 2 nm).

**Figure 6:**
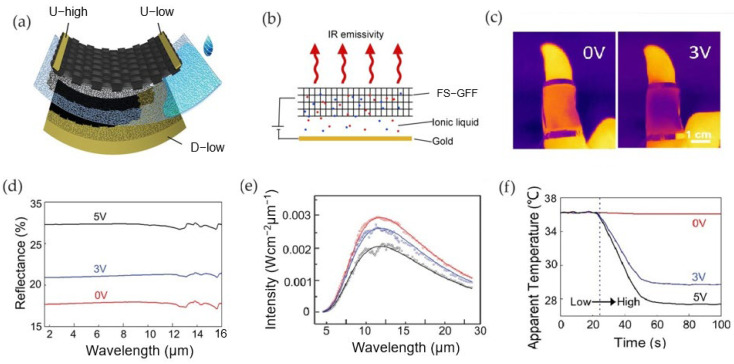
Cui et al. [70]. Adjustable infrared camouflage flexible textile device based on FS-GFF. (**a**) schematic of the device; (**b**) ionic intercalation; (**c**,**d**) Reflectance and infrared emission spectra; (**e**) apparent temperature change versus different voltages; (**f**) infrared camouflage ability images. Adapted with permission from Ref. [70]. Copyright 2022 American Chemical Society.

The device was assembled using FS-GFF as the top layer, a fabric separator as the middle layer, sputtered gold as the back electrode, and 1-butyl-3-methylimidazolium hexafluorophosphate (BMIMPF_6_) as the ionic liquid electrolyte (Figure 6a). By applying a voltage from 0 to 5 V, ionic motion was induced (Figure 6b). 

By applying an increasing bias, the infrared reflectance of the device increases, the Fermi energy increases, and the optical conductance of FS-GFF changes, indicating that the intercalation of ions occurred. Also, the infrared reflectance of the device improved from 21% to 32% when a 5 V voltage was applied (Figure 6c). Correspondingly, the infrared emission of FS-GFF at different voltages was measured, as shown in Figure 6f, resulting in a modulation of the infrared emissivity between 0.79 and 0.68.

At ≈3 V, the apparent temperature is reduced (i.e., the infrared emissivity is reduced) (Figure 6e,f). The FS-GFF-based device was able to shield the target from infrared detection, being suitable as an IR camouflage device.

**Figure 7:**
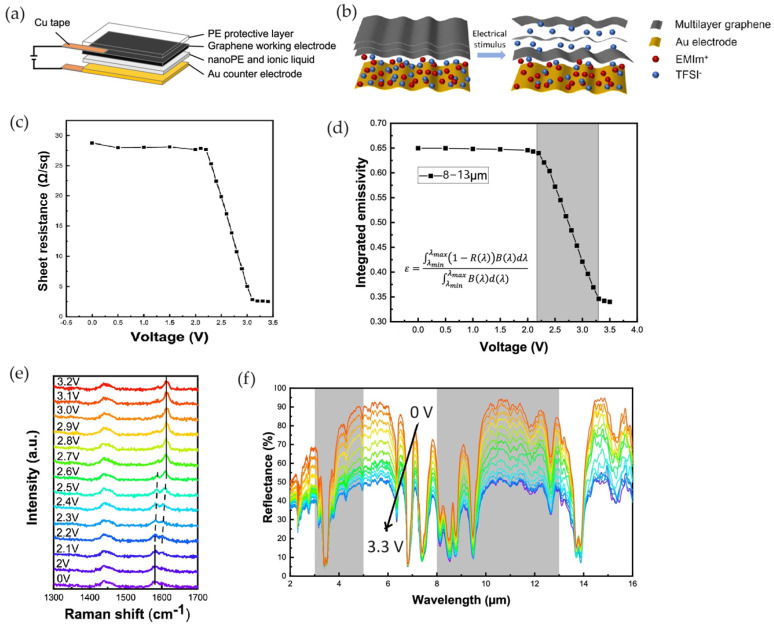
Li et al. [71]. Schematic of the graphene-based soft actuator (**a**) and ionic intercalation (**b**); (**c**) sheet resistance modulation, (**d**) emissivity modulation; (**e**,**f**) Raman and reflectance spectra measured at different applied voltages. Adapted with permission from Ref. [71]. Copyright 2022 American Chemical Society.

Li et al. [71] developed a graphene-based smart surface having a dual function of actuation and spectral regulation. The device consisted of a three-layer electrochemical structure made of an electroactive graphene working electrode, a polyethylene (PE) separator containing a non-volatile ionic liquid EMIM-TFSI, and an Au counter electrode (Figure 7a). 

The device achieved emissivity regulation from 0.65 to 0.35 detected within a specific voltage from 0 to 3.3 V. The reduction in emissivity was coupled with a reduction in the sheet resistance of the material. Indeed, measurements of the in situ sheet resistance indicated that it was maintained at ∼28 Ω/sq below 2.2 V and decreased sharply to ∼3 Ω/sq when the voltage increased up to 3.1 V, consistent with the integrated emissivity (Figure 7c,d).

In situ Raman spectra were obtained under a working voltage range of 0–3.2 V. Between 2.2 and 2.6 V, the G peak splitting demonstrated the formation of the stage reaction of ion intercalation, which resulted in the G peak shifting from 1582 cm^−1^ to approximately 1610 cm^−1^, as shown in Figure 7e. Furthermore, the MIR reflectance spectrum was almost flat at ∼40% and did not increase until 2.3 V, then slowly increased as the voltage increased to 3.3 V. The ions intercalated into the graphene layers caused the energy level to change and the optical conductivity to increase, enhancing the graphene electrode layer’s reflectance.

A capacitance of 8.14 F/g was evaluated by the cyclic voltammetry test, indicating that the device works as an electrochemical capacitor. Electrochemical impedance spectroscopy (EIS) tests showed different behaviors according to the voltage bias: at stage one (1.0 V) the diffusion of the ions was completed; after 2.3 V the device exhibited a first capacitance associated with the graphene electrode layer; after 3.3 V, a second capacitance was found due to ion aggregation in the gap between the active layer and the separator. 

Yu et al. [67] studied the effect of ionic liquid on the long-term performance of graphene multilayer optoelectronic devices within a broad infrared wavelength range. 

**Figure 8:**
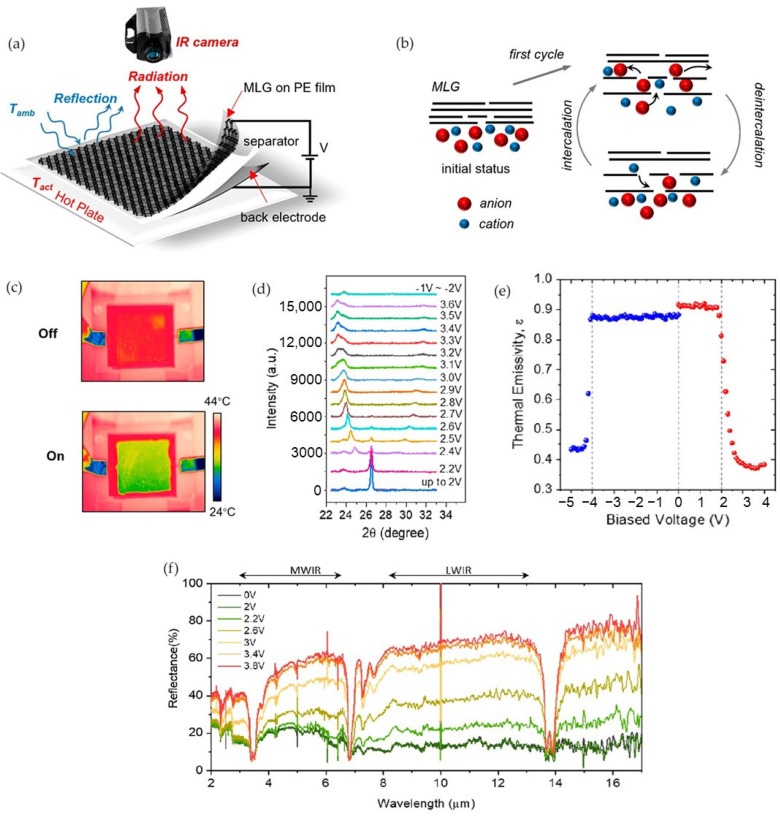
Yu et al. [67]. Schematic structure of device (**a**). Ionic intercalation process (**b**). Apparent temperature variation by IR camera (**c**). XPS spectra (**d**). Emissivity modulation (**e**). Reflectance spectra (**f**). Adapted with permission from Ref. [67]. Copyright 2023 American Chemical Society.

The schematic structure of the device is shown in Figure 8a and consists of four layers: laminated layers of MLG film on a polyethylene membrane, a porous separator (Whatman 105 lens cleaning tissue, 2105-841) soaked with the ionic liquid [AMIM]^+^[TFSI]^−^, and the stainless steel back electrode. 

The device was prepared by coupling a low-density polyethylene membrane, which is transparent in the IR window of interest, and the MLG layer in order to avoid the rupture of MLG layers during the handling processes.

The authors investigated the performance of the device under positive and negative voltage biases with the aim of observing both the intercalation and deintercalation phenomena. They observed that the threshold voltage depended on many factors, such as the material of the back electrode, the size of ionic liquid particles, and the thickness of the ionic liquid layer, which was 2.2 V for the device at positive voltages with anion intercalation. 

Under a positive bias voltage (from 0 V to 3.8 V), the MLG film becomes hole-doped due to anion insertion within MLG defects. During the CVD graphene layer growth process, defects such as gaps and grain boundaries may be formed, resulting in favorable access areas for ions. Once intercalated ions penetrate, they diffuse between the layers of MLG, expanding the interlayer spacing between the sheets and leading to a structural change in the MLG. The effect of intercalation results in a reduction in the apparent temperature and in the infrared emissivity that changes from 0.92 at 0 V to 0.37 at 3.8 V (Figure 8c–e).

However, in situ XPS spectra indicated a dual intercalation of both anions and cations. Figure 8d shows that, starting from 2.2 V, the original MLG structure is gradually disrupted by intercalating the [TFSI]^−^ anion, since new diffraction peaks emerge. Intercalated ions then expand the interlayer spacing between graphene sheets and lead to a structural change for the MLG.

When applying a negative bias, voltage deintercalation occurs, with an emissivity recovery of 92.3%. The device is not fully brought back to its initial state; some ions remain trapped between graphene layers. The device can remain at the intercalated state with lower bias voltage (2.6 V), and it almost restores to its initial state at low inverse voltages (−2 V).

**Figure 9:**
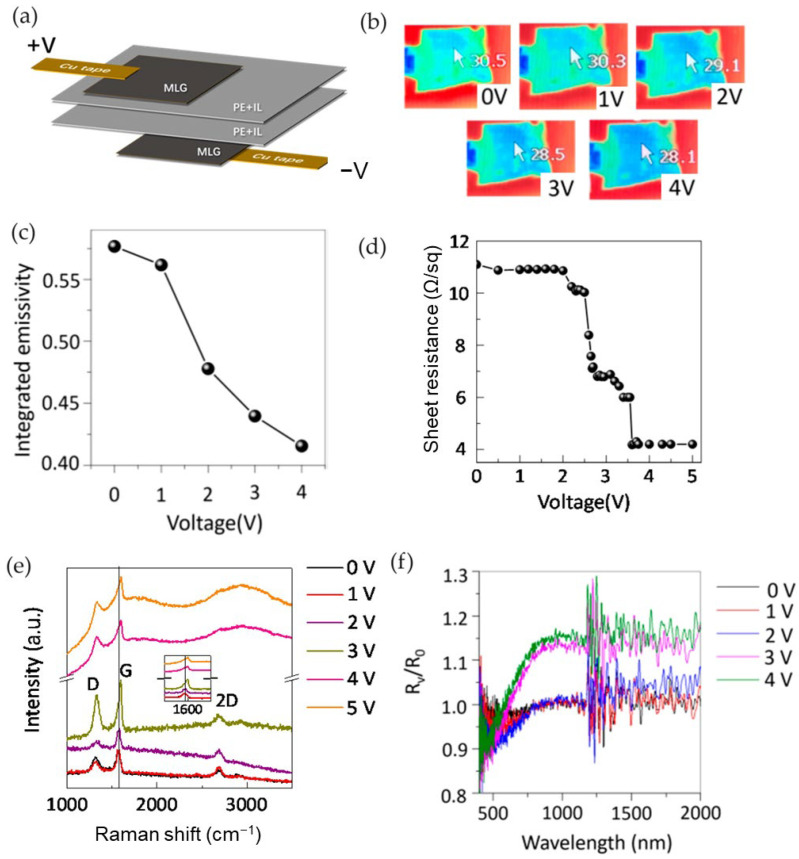
Zhao et al. [72]. Schematic structure of device (**a**). Apparent temperature variation by IR camera (**b**). Emissivity (**c**) and sheet resistance modulation (**d**). Raman (**e**) and reflectance (**f**) spectra. Adapted with permission from Ref. [72]. Copyright 2019 MDPI Journals.

Zhao et al. [72] investigated the performance of a thermal camouflage device using MLG both as an active layer and back electrode. The authors fabricated an infrared tunable device by attaching two MLG layers coated on porous polyethylene membranes together, with the MLG facing outward. About 50 μL of [DEME]^+^[TFSI]^−^ ionic liquid was injected between the two membranes (Figure 9).

The device was tested within the electrochemical window of the ionic liquid in the bias voltage range of 0 to 4 V. When the bias voltages exceeded 5 V, the graphene surface turned dark black and could not be reversed, likely due to the oxidation of surface graphene, which becomes sensitive after heavy doping. Figure 9b shows that the apparent temperature of the device decreased by 2.4 °C at 4 V, indicating that the device’s emissivity was suppressed by ionic liquid intercalation (Figure 9c). At 2 V, ionic liquid intercalation reduced emissivity from 0.57 to 0.41, while the sheet resistance of MLG varied from 11 to 4 Ω/sq above 3.5 V (Figure 9d).

Owing to the ionic liquid intercalation, the Fermi level of graphene shifted to a higher energy level, resulting in decreased emissivity/absorption and in increased transmittance. Reflectance measurements showed an increase in reflectance above 3 V (Figure 9f). The relative reflectivity of surface graphene increased by 0.1 at 4 V, indicating reduced absorption/emissivity above 3 V. 

The intercalation process of surface multilayer graphene was also monitored by in-situ Raman measurements. The Raman spectrum does not change for a bias lower than 2 V; exceeding 3 V, the G and D peaks increase in intensities and shift from 1580 to 1603 cm^−1^ (Figure 9e). The increased intensity of the G peak is due to the doping effect of the intercalation of ions, whereas the increased D peak intensity indicates the presence of defects in the graphene layers during the intercalation phase.

**Figure 10:**
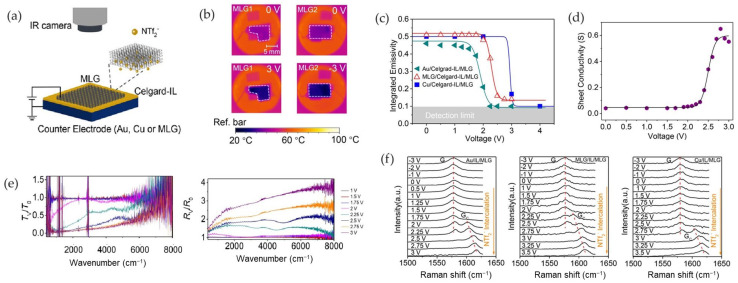
Sun et al. [44]. (**a**) Schematic structure of device and test apparatus. (**b**) Thermal images of the device MLG/Celgard-IL/MLG at different voltages. (**c**) Emissivity modulation. (**d**) Sheet DC electrical conductivity with applied voltage for Au/Celgard-IL/MLG sample; (**e**) Reflectance/transmittance spectra for MLG/Celgard-IL/MLG device; (**f**) Raman spectra. Adapted with permission from Ref. [44]. Copyright 2019 American Chemical Society.

Sun et al. [44] investigated the performance of a device based on MLG and intercalated by the ionic liquid (HMIm[NTf_2_]) on both rigid and flexible substrates. The authors fabricated three different devices by changing the counter electrode: they used Au, Cu, and MLG as back electrodes (Figure 10a). 

In the case of double MLG electrodes, an 18 °C decrease in the apparent temperature was found for a voltage of 3 V (Figure 10b). 

By comparing the three devices, the same decrease in emissivity was found (Figure 10c). However, a difference in the threshold voltage was observed. In the case of samples with Au and Cu as electrodes, the emissivity reduced by nearly 80% (from 0.5 to 0.1), while a lower reduction was found in the case of the MLG/MLG device. At the same time, the electrical conductivity of the MLG film increased by an order of magnitude as the applied voltage increased from 0 to 3 V (Figure 10d). Also, the decrease in the emissivity was accompanied by a corresponding increase in reflectance and decrease in transmittance (Figure 10f). 

To further confirm the intercalation process in the MLG active layer, Raman spectra were collected under an applied voltage. As the voltage increased to the intercalation voltage of 2 V, a shift in the Raman peak, named G_C_, occurred at ∼1601 cm^−1^. The coexistence of both G and G_C_ resulted from the partial intercalation of the MLG film, whereas the disappearance of the G peak indicated the absence of undoped MLG film and the formation of a stage 2 phase. The G_C_ peak appeared at different voltages for Au (2.0 V), MLG (2.25 V), and Cu (2.75), indicating that the counter electrode influenced the threshold voltages.

Finally, the authors investigated the effect of the amount of IL injected in the membrane on the performance of the device with an Au electrode, showing that both the response time and modulation depth were not significantly affected by the amount of IL.

### 2.3. Thermal Camouflage Devices Based on vdW Graphene Layer

Li et al. [73] fabricated a graphene-based electrochemical device by using a large-area vdW graphene film (vdWGRf) as the active layer. The authors developed a technique to build graphene films on various polymer substrates by simple mechanical adhesion without any additives. Graphene nanosheets were transferred to the substrate with a layer-by-layer structure. Firstly, the nanosheets were attached to a synthetic rubber carrier and then deposited on a PTFE substrate by dragging. By repeating the dragging–adhesion–separation process, graphene nanosheets remained on the PTFE substrate by vdW force until a compact coating was formed. The transfer process and the formation of a layer-by-layer coating was possible only if the surface energies of the polymer substrates were similar to those of the graphitic nanoplatelets. 

**Figure 11:**
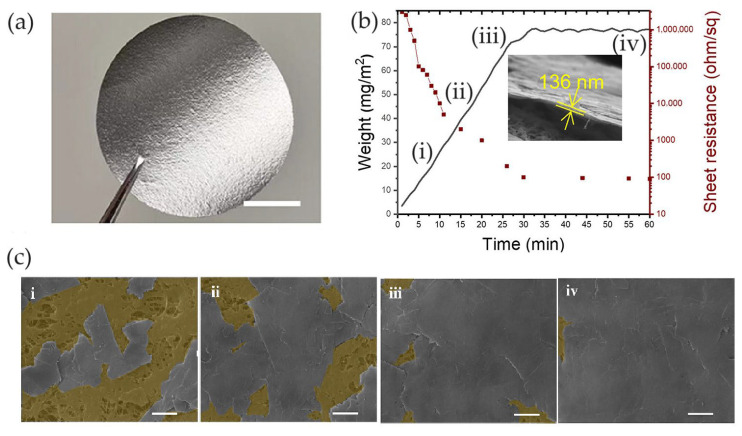
Li et al. [73]. (**a**) vdWGRf on PTFE membrane; (**b**) weight variation and sheet resistance of the PTFE-based vdWGRfs with increasing preparation time and (**c**) coating aspect at different deposition times. Caption (**i**–**iv**) refers to the deposition steps. Adapted with permission from Ref. [73]. Copyright 2023 John Wiley & Sons.

These coatings exhibited a strong metallic cluster, associated with the layer-by-layer stacking structure and high electrical properties (Figure 11a). The process allowed for the formation of samples with a thickness of around 136 nm, which corresponds to a sheet resistance of 92.8 ± 4.6 Ω/sq (Figure 11b).

**Figure 12:**
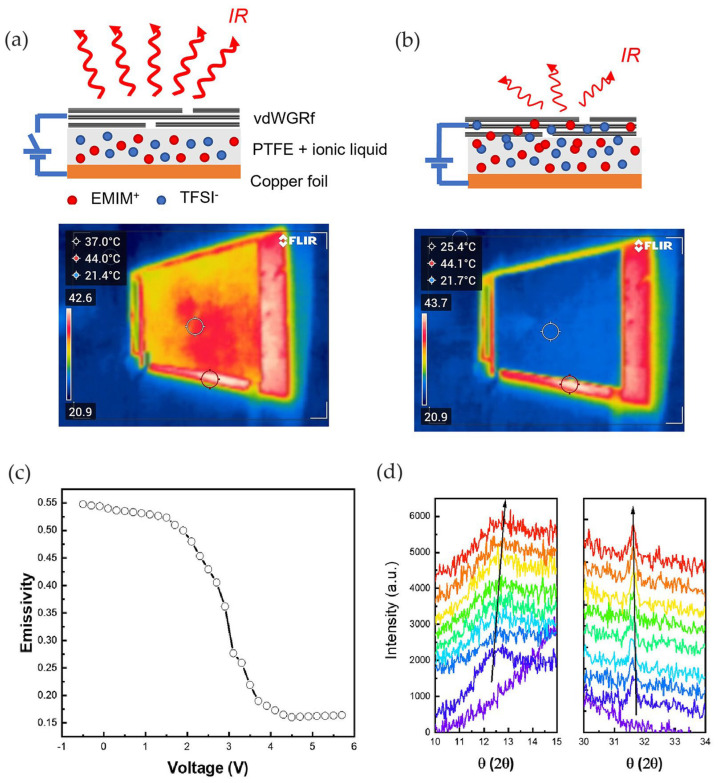
Li et al. [73]. Schematic structure of device and test apparatus before and after actuation and thermal images of the device at (**a**) 0 V and (**b**) 4 V. (**c**) Emissivity modulation. (**d**) XRD spectra at increasing applied voltage from 0 V (purple line) to 4 V (redline). Adapted with permission from Ref. [73]. Copyright 2023 John Wiley & Sons.

The electrochemical device of 3 cm × 3 cm size was composed of a thin GNP layer of 136 nm deposited on a PTFE membrane as the active layer and a copper conductive tape as the counter electrode layer; 20 μL of [EMIM][TFSI] ionic liquid electrolyte was used in this device (Figure 12a).

The working principle of this device is equal to that of devices made of an MLG active layer. When a voltage bias is applied to the electrochemical device, the ions intercalate into the GNPs, modifying the Fermi energy and carrier density, thus altering the active layer’s optical properties (Figure 12b). The authors identified the efficient working voltage window of the device between 0 and 4.4 V and observed a device failure at 5 V. At 4 V, the apparent IR temperature decreased, matching that of the background. The device showed an excellent thermal regulation capability of suppressing 90% of radiative heat transfer and reducing the emissivity from 0.55 to 0.10 (Figure 12c).

In situ X-ray diffraction (XRD) was performed to check the intercalation phenomenon. It showed the appearance of two new peaks (00n + 1) and (00n + 2) as a signal of TFSI anions intercalating into GNPs (Figure 12d).

Since the device showed a capacitor nature, a slightly negative voltage of −0.5 V was applied to the device in order to release the accumulated cations on the vdWGRf. The stability of the regulator was tested in repeated voltage cycles, showing that the device exhibited was also able to regulate the emissivity with the same depth after 300 cycles. Moreover, the device demonstrated an electrochemical activity as shown by the quasi-rectangle shapes and instantaneous current response to voltage reversal during cyclic voltammetry. The specific capacitance of the device was calculated, with a maximum value of 16.95 F/g at a scan rate of 0.002 V/s. 

### 2.4. Thermal Camouflage Devices Based on Graphene Aerogels (GAs)

Other nanostructures have been developed to enhance heat absorption and blend the target’s IR signature with the surrounding environment. Weng et al. [74] proposed graphene aerogels (GAs) with high porosity used as electrodes in an infrared emissivity modulator. The aerogel was made of MLG layers assembled in a porous continuous three-dimensional structure.

**Figure 13:**
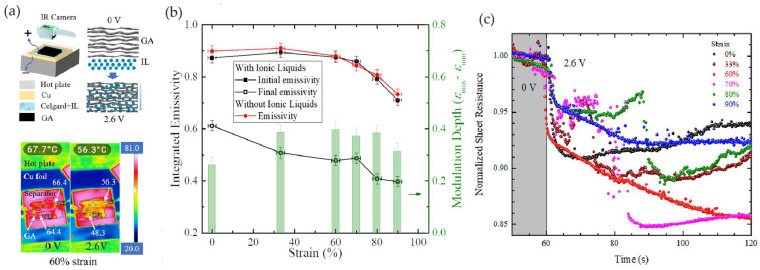
Weng et al. [74]. (**a**) Schematic structure of device and IR images of GA before and after applying a bias voltage. (**b**) Emissivity modulation of GA as a function of strain. (**c**) Sheet resistance of GA with different strains vs. bias voltage. Adapted with permission from Ref. [74]. Copyright 2022 APS Publications.

The infrared emissivity modulator consisted of a GA active layer, a PP-PE-PP separator of 25 µm thickness injected with [HMIm][NTf_2_] ionic liquid, and a polished Cu back electrode (Figure 13a). 

The apparent temperature of graphene aerogel (GA) under 60% strain, as measured by a thermal camera, decreased by 11.4 °C when a voltage bias of 2.6 V was applied (Figure 13a). The lowest emissivity of the samples is shown in Figure 13b. Initially, the emissivity of GA at 0 V remained nearly unchanged (approximately 0.88) for strains below 60%, then quickly dropped to 0.71 with a strain of 90%. A set of GAs with the same strains but without ionic liquids (ILs) showed similar results (marked as red dots in Figure 13b), indicating that the change in initial emissivity was likely due to strain, rather than the presence of ILs. Ion intercalation in GA occurs at 2.6 V: some anions adsorb on or intercalate into graphene layers, transferring charges to the graphene carbons and significantly increasing the charge density of GA. This raises the Fermi level, altering the transitions of charge carriers and thus decreasing the emissivity of the device (Figure 13b) and decreasing the sheet resistance of the graphitic layer (Figure 13c).

Different from the other graphene-based materials described in the previous sections, the porous structure of GA facilitates IL transport within the electrode and provides greater resilience to ion intercalation, resulting in less structural damage. Additionally, the charge transferred to graphitic structures enables both anion intercalation and anion adsorption on the three-dimensional network structures formed by MLG in GA. This enhances the device’s lifetime while achieving a comparable modulation depth.

## 3. Discussion

**Figure 14:**
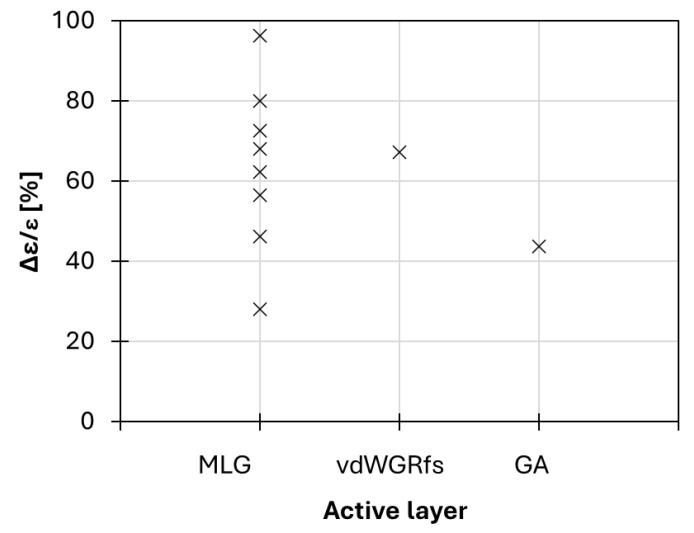
Emissivity modulation of thermal adaptive camouflage devices in literature.

The emerging field of 2D nanomaterials offers numerous opportunities for advancements in thermal camouflage techniques. Typically, camouflage material design alone cannot protect targets from multispectral detection systems. Therefore, there is an urgent need for multispectral camouflage technologies that cover the IR, visible, and/or radar wavelength ranges. The electromagnetic properties required for stealth materials differ across bands: radar stealth materials need high absorption and low reflectivity, whereas IR stealth materials require low emissivity and high reflectivity within the atmospheric windows of 3–5 μm and 8–14 μm. This paper reviews the advancements in IR camouflage using 2D graphene and graphene-like material. In this review, the application of graphene-based surfaces for temperature control has been reported by analyzing the literature. As reported in Table 3, different adaptive thermal camouflage devices have been realized by changing the active layer, the separator, the ionic liquid, and the back electrode to obtain emissivity variation with voltage bias. Concerning the characteristics of the graphene-based active layers, a large part of the articles uses thicknesses of about 100 nm with sheet resistance of a few tens of Ohms per square. Specifically, 80% of works were based on MLG as active material (Figure 14). Only in recent years have new systems been investigated as an alternative to MLG to simplify the production process. Regardless, currently, the performances of the reported new materials seem to be lower in terms of apparent temperature variation and response times (as shown in Table 3 and Figure 14). However, direct comparison among the analyzed different systems is complex due to the differences in device architectures and real temperatures (T_real_) applied during the measurements. For example, a large number of the experiments have been carried out on MLG-based devices setting T_real_ higher than 60 °C. This condition could emphasize the variation between the samples and the ambient temperature, as reported in [42]. The performance of the device in terms of temperature modulation range depends on the difference between the system and the background temperatures. 

In general, the intercalation process is obtained for a voltage threshold higher than 2 V, and the identified wider working window is in the range of 0–5 V. At higher voltages, stability problems of the devices occur. Mainly all the intercalations have been carried out with ionic liquids containing [TFSI]^−^ or [NTf_2_]^−^ as anions. Instead, the most used cations are [HMIm]^+^ and [EMIM]^+^, which cover 72% of the works together. All the reported devices showed good emissivity modulation for dynamic thermal camouflage (about 0.35 unit of variation) in a short time (a few seconds), being promising for military applications. Works in the literature showed that objects with modulable emissivity are able to perform dynamic thermal camouflage under IR detection systems, also for in-movement targets or background temperature changes. To date, IR emissivity tuning strategies to achieve dynamic thermal camouflage with graphene are based on electrical and strain modulation. Other methods, such as phase change modulation, optical modulation, chemical modulation, and wetting modulation, have received less attention in the context of graphene. These unexplored strategies warrant further investigation to enhance graphene’s potential applications in thermal camouflage. 

To direct the development of such technology toward real applications, several elements and performance indicators should be considered to compare and select among different EC materials. EC materials over other chromogenic materials are an obvious choice since, in most cases, external biasing is the preferred way to control a device operation. Of utmost importance is the time response of the device since, in many scenarios, switching between different emissivity states should occur in real time. Moreover, the available emittance modulation range should be large to guarantee a wide operation range for the device; hence, the physicochemical process driving electrochromism should significantly change the surface state. Furthermore, certain applications require the possibility to modulate emittance over the full mid-IR spectrum, so the surface should be engineered to have broadband mid-IR absorption. Instead, resonant designs employing, e.g., nanostructures and metasurfaces could be useful when working at specific IR wavelengths. The overall thickness and compatibility with flexible substrates are also important assets in dynamic camouflage applications. Finally, the temperature range of operation, device wearing and cyclability, resistance to other external stimulation (mechanical shocks, irradiation, …), lightness, fabrication cost, etc., should be taken into account in developing thermal camouflage technology on a large scale. Therefore, there are still open challenges to be addressed for this technology, such as, to name a few, fabrication of reliable, large-area devices at reasonable cost, extensive testing for the application in real-world scenarios, and qualification of materials.

## 4. Conclusions

The growing sector of 2D nanomaterials is creating new opportunities for developing adaptive coatings for IR radiation management, including thermal camouflage and radiative cooling technologies. Among these materials, graphene and graphene-like materials stand out due to their highly customizable mechanical, thermal, and electro-optical properties, and multi-band compatibility, which are unmatched by other platforms. Graphene and its composites are expected to attract, in the future, thermal camouflage and multi-spectrum stealth applications. 

This review has highlighted the development of camouflage materials based on voltage modulation of the emissivity and reflectivity of graphene-based layers. This technology allows for the design of new camouflage devices that can change the appearance of hot surfaces to cold surfaces in thermal imaging systems. The key requirement in this application is to suppress the emissivity of the active surface, blending the object into the background. The change in emissivity makes the active surface appear colder until it matches the background temperature. The infrared thermally adaptive device operates by electro-modulating the IR absorptivity and emissivity of a graphene surface through the reversible intercalation of non-volatile ionic liquids. Under a voltage bias, the ionic liquid intercalates into the graphene layer, increasing its charge density. Consequently, the Fermi level shifts to higher energies, suppressing IR absorption and lowering the emissivity of the graphene electrode.

However, further work is needed to design and synthesize 2D graphene materials for developing multiband-compatible stealth. One of the main issues is oxidation at high temperatures, which can significantly degrade the material properties and their thermal camouflage capabilities, leading to camouflage failure. Therefore, specific studies should focus on the stability of graphene and graphene-like materials under high-temperature thermal camouflage conditions.

This review aims to provide guidelines for selecting materials for next-generation IR stealth applications and to inspire researchers from various fields to explore and discover new performance characteristics.

## Figures and Tables

**Table 1:** Pros and cons of thermal camouflage materials.

Materials for Thermal Camouflage	PROS	CONS	Ref.
Metallic Powders	Emissivity reduction by topological modification of the surface; Low cost	Rigid structure; High reflectivity; Oxidation; No dynamic camouflage	[7,8,9,10]
Metal-oxide	Excellent stability; Dynamic camouflage; Multispectral stealth	High cost; High emissivity	[16,17,18,19]
Metamaterials	Low emissivity modulation; Dynamic camouflage; Multispectral stealth	High cost	[20,21]
Thin metallic film	Very large emittance modulation	Slow response time; Limited lifetime and cyclability	[22,23,24,25,26]
MXenes-based materials	Emissivity reduction; lightweight; microwave absorption capacity; slight sensitivity to temperature	Dependence on surface morphology, structure, and functional groups of nanoparticles	[13]
Graphene and graphene-like materials	Dynamic camouflage; Multispectral stealth	High emissivity	[5,27,28]

**Table 2:** Separators used for fabricating adaptive camouflage devices.

Composition	PP-PE-PP	PE	PTFE	Cellulose Paper
Thickness (µm)	25	20		15
Average pore size (µm)	0.070–0.150	-		0.22
Porosity (%)	39	-		-
Temperature stability (°C)	135–163	-		150–180 °C
Water wettability	Hydrophobic	Hydrophobic	Hydrophobic	Hydrophilic
Electrolyte wettability	Good	Good	Not well	Good

**Table 3:** List of studies on thermal adaptive camouflage devices (R_s_ = Sheet resistance; V_th_ = Threshold voltage; T_real_ = Real temperature of the device; T_app_ = Apparent temperature of the active surface; t_R_ = Response time).

Active Layer	Thickness (nm)	Electrolyte (Volume)	Separator	Back Electrode	Bias (V)	R_s_ (Ω/sq)	V_th_ (V)	T_real_ (°C)	T_app_ (°C)	ε (-)	t_R_ (s)	Ref
MLG	34	[DEME]^+^[TFSI]^−^	PE	Au	0 to 3.5	33–0.6	1.5 V	55°	N.A.	0.76–0.33	N.A.	[41]
MLG	50–70	[DEME]^+^[TFSI]^−^ (50 µL)	PE	MLG	0 to 4.0	11–4	2.0 V	35°	~31°–28°	0.57–0.41	<1 s	[72]
MLG	-	[EMIm]^+^[NTf_2_]^−^	PP-PE-PP	Cu	0 to 4.0	N.A.	2.0 V	65°	~48°–32°	0.54–0.02	N.A.	[69]
MLG	90–100	[HMIm]^+^[NTf_2_]^−^	PP-PE-PP	Au	0 to 4.0	N.A.	2.0 V	65°	~50°–32°	0.47–0.10	~1.8 ÷ 1.9	[44]
MLG	90–100	[HMIm]^+^[NTf_2_]^−^	PP-PE-PP	MLG	0 to 4.0	N.A.	2.5 V	65°	~50°–30°	0.51–0.14	~1.7	[44]
MLG	90–100	[HMIm]^+^[NTf_2_]^−^	PP-PE-PP	Cu	0 to 4.0	N.A.	3.0 V	65°	N.A.	0.50 –0.10	N.A.	[44]
MLG	-	[AMIM]^+^[TFSI]^−^	Lens cleaning tissue	Stainless steel	0 to 3.8	50–N.A.	2.2 V	N.A.	~41°–31°	0.85–0.33	N.A.	[67]
MLG	N.A.	[EMIM]^+^[TFSI]^−^ (20 µL)	PE	Au	0 to 3.3	28–3	2.7 V	N.A.	N.A.	0.65–0.35	N.A.	[71]
FS-GFF	90	[BMIM]^+^[PF_6_]^−^	Cellulose	Au	0 to 5	50–N.A.	3.0 V	N.A.	~36°–27°	0.79–0.68	~25	[70]
vdWGRfs	136	[EMIM]^+^[TFSI]^−^ (20 µL)	PTFE	Cu	0 to 5.5	98–N.A.	3.0 V	38°	~31°–27°	0.55–0.18	~6	[73]
GAs 20% porosity	60,000	[HMIm]^+^[NTf_2_]^−^	PP-PE-PP	Cu	0 to 2.6	N.A.	2.6 V	69°	~68°–56° (@60% strain)	0.71–0.40	~180	[74]

## Data Availability

Not applicable.
